# School-Based Intervention to Improve Healthy Eating Practices Among Malaysian Adolescents: A Feasibility Study Protocol

**DOI:** 10.3389/fpubh.2020.549637

**Published:** 2020-09-22

**Authors:** Shooka Mohammadi, Tin Tin Su, Muhammad Yazid Jalaludin, Maznah Dahlui, Mohd Nahar Azmi Mohamed, Angeliki Papadaki, Russell Jago, Zoi Toumpakari, Hazreen A. Majid

**Affiliations:** ^1^Department of Social and Preventive Medicine, Faculty of Medicine, Centre of Population Health, University of Malaya, Kuala Lumpur, Malaysia; ^2^South East Asia Community Observatory (SEACO), Jeffrey Cheah School of Medicine and Health Sciences, Monash University Malaysia, Subang Jaya, Malaysia; ^3^Department of Paediatrics, Faculty of Medicine, University of Malaya, Kuala Lumpur, Malaysia; ^4^Department of Sports Medicine, Faculty of Medicine, University of Malaya, Kuala Lumpur, Malaysia; ^5^Centre for Exercise, Nutrition and Health Sciences, School for Policy Studies, University of Bristol, Bristol, United Kingdom; ^6^Department of Nutrition, Faculty of Public Health, Universitas Airlangga, Surabaya, Indonesia

**Keywords:** Malaysian adolescents, school canteen, dietary habits, eating practices, school-based

## Abstract

**Introduction:** School environments can influence students' dietary habits. Hence, implementing a healthy canteen intervention programme in schools is a recommended strategy to improve students' dietary intake. This study will evaluate the feasibility of providing healthier food and beverage options in selected secondary schools in Malaysia by working with canteen vendors. It also will assess the changes in food choices before and after the intervention.

**Methods:** A feasibility cluster randomised controlled study will be conducted in six secondary schools (intervention, *n* = 4; control, *n* = 2) comprising of rural and urban schools located in Selangor and Perak states in Malaysia. Four weeks of intervention will be conducted among Malaysian adolescents aged 15 years old. Two interventions are proposed that will focus on providing healthier food options in the canteen and convenience shops in the selected schools. Interventions 1 and 2 will entail training the canteen and school convenience shop operators. Intervention 2 will be applied to subsidise the cost of low energy-dense *kuih* (traditional cake), vegetables, and fruits. The control group will continue to sell the usual food. Trained dietitians will audit the canteen menu and food items sold by the school canteen and convenience shops in all schools. Anthropometric measurements, blood pressure and dietary assessment will be collected at baseline and at the end of 4-week intervention. Focus group discussions with students and in-depth interviews with headmasters, teachers, and school canteen operators will be conducted post-intervention to explore intervention acceptability. Under this Healthy School Canteen programme, school canteens will be prohibited from selling “red flag” foods. This refers to foods which are energy-dense and not nutritious, such as confectionery and deep-fried foods. They will also be prohibited from selling soft drinks, which are sugar-rich. Instead, the canteens will be encouraged to sell “green flag” food and drinks, such as fruits and vegetables.

**Conclusion:** It is anticipated that this feasibility study can provide a framework for the conception and implementation of nutritional interventions in a future definitive trial at the school canteens in Malaysia.

## Introduction

There is a concern about the growing prevalence of obesity and unhealthy eating habits among adolescents (1, 2). Obesity and unhealthy diets are associated with chronic diseases, such as cardiovascular disease (3, 4). Promoting healthy eating among adolescents has become an important public health and research priority because the incidence of obesity and overweight among adolescents continues to increase and tends to persist into adulthood (5, 6). Skipping breakfast and high consumption of energy-packed foods are considered among risk factors that lead to overweight and obesity in adolescents (7, 8). A National Health and Morbidity Survey (NHMS) reported that, among Malaysians aged 10–17, the prevalence of obesity had risen from 5.7% in 2011 to 11.9% in 2015 (9–11).

An unhealthy diet contributes significantly to weight gain and obesity. Studies have shown that the quality of the diet declines when children enter adolescence (12). The consumption of fruit, vegetables, and milk decrease, while the intake of sugar-sweetened beverages and confectionery increases through adolescence and early adulthood (13). Fruits and vegetables are key foods that will reduce fat, energy density and increase fibre that are related to lower cardiovascular disease risk in adolescents (14). A cohort study in the UK revealed that childhood dietary habits, namely low fibre intake and consumption of high fat and energy-dense food, are linked to increased adiposity in adolescence (15). Therefore, a healthy diet can lower the risk of obesity among younger people (15). A longitudinal study (MyHeARTs) in Malaysia suggested that students who live in rural areas consumed more sugar, cholesterol, and energy compared with their peers in urban schools (16). Several observational studies (16, 17) have been conducted to understand the dietary patterns among Malaysian adolescents. The findings show that Malaysian adolescents are prone to consume unhealthy foods (18), and have unhealthy eating behaviours (19). The current available data on school-based interventions are not culturally relevant to schools and students in Malaysia. Thus, it is important to design a dietary intervention programme that is evidence-based and culturally relevant for Malaysian adolescents and can be delivered and evaluated in the Malaysian setting. Malaysian foods are influenced by the preparation methods that differ across the Malay, Indian, and Chinese ethnicities. Different ethnic groups have different cuisines and variety of food preparation methods. This leads to different food preferences and eating habits among the ethnicities in Malaysia. Hence, it is important to incorporate cultural differences into designing dietary interventions for better acceptance of healthy foods among school-going adolescents.

Adolescents' dietary behaviour is likely to be strongly influenced by environmental factors (20, 21). The school food environment is often considered as a target for nutritional intervention as school children consumed ~40% of their daily dietary intake at schools (22). Two promising school food environment policies have been introduced by providing fresh fruits and vegetables (F&V) in addition to restricting the sale of sugar-sweetened beverages (SSBs) (23–26). It has been shown that a higher intake of F&V and reduced consumption of SSBs (sodas, sports, and energy drinks) may be beneficial for children by reducing the incidence of cardiometabolic disease (27). Previous interventions conducted in schools in Norway, the Netherlands and US have tried to provide free or subsidised fresh fruits and vegetables, usually as snacks in addition to school meals (28–30).

Interventions targeting the consumption of beverages is essential as many Malaysian adolescents consume large amounts of SSBs, milk, coffee, cordials, and fruit-flavoured drinks with added sugars (31). An 18-month trial among 641 normal-weight children in the Netherlands revealed that replacing SSBs with zero-calorie beverages decreased weight gain and fat accumulation in normal-weight children. This suggests that reducing SSBs intake can cause an impact on body weight in children (32). A systematic review indicated that school-based education programmes focusing on the reduction of SSBs intake with follow-up modules could provide chances for conducting effective and sustainable interventions (33). In addition, by modifying the school environment (e.g., providing water and reorganization of beverages by promoting less SSBs) and having peer-support group to promote health educational programmes could improve their effectiveness (33). Home delivery of more proper drinks has a major effect on the reduction of SSBs consumption and related weight loss (33).

Several initiatives have been implemented in Malaysia and across the world to ensure adherence to established nutritional guidelines and standards, healthy canteen policies, and strategies for all foods served in school canteens (34–36). However, the impact of these programmes and their long-term sustainability are unclear. Addressing this gap is particularly important in Malaysia, where the school is an important provider of two main meals (breakfast and lunch) (37). In a recent systematic review about Malaysian adolescents' dietary intake, there was a small difference in dietary patterns according to ethnic diversity, which indicated that Malays had higher SSBs intake and lower diet quality than Chinese and Indian adolescents (21). Furthermore, Malay adolescents preferred Western-based and local-based dietary patterns while Chinese adolescents intended to follow a healthy-based food pattern, which may reflect the effect of socio-cultural diversity on food preferences (21).

In Malaysia, the schoolchildren purchased food from the canteen and *koperasi* (school convenience shop) which are run by the canteen operators and school administration, respectively. The *koperasi* sells mostly energy-dense snacks and beverages. The staple foods usually was sold at the canteen such as noodles, fried rice, nuggets, and fried chicken, energy-dense traditional cakes (e.g., *Rempeyek, Kuih Peneram, Kerepek Ubi Kayu, Kuih Baulu*, etc.), and SSBs.

Environmental interventions are suggested as more likely to be effective in producing behavioural change (38). Evidence from systematic reviews, which mostly comprised studies implemented in the US, indicated that school-based strategies can be effective in cultivating healthy eating habits and food purchasing behaviours among school children (39–41). These reviews found that by widening choices of healthy foods, limit the sales of junk foods, and affordable price of healthy food options may help to increase intake of fruits and vegetables and reduced intake of saturated fat among school children (39–41). Nevertheless, few studies explored the extent to which school canteens implemented promotion and pricing strategies to promote the purchasing of healthy foods and drinks (42). Studies on food environments in schools have concentrated on reporting the accessibility of healthy foods sold in canteens, in school lunch programmes, or sold by using vending machines with limited information on other practices (42–44). Therefore, the purpose of this study is to evaluate the feasibility of delivering a new programme that will promote the availability of healthy food and drink options, especially at school canteens and school convenience shops in Malaysian secondary schools. We also seek to examine the feasibility of collecting data to assess this intervention approach to determine if a larger evaluation is warranted.

### Aims

The general aim of this study is to assess the feasibility of an intervention programme to improve availability of healthy food items at the school canteen and convenience shop and promote healthy eating practices among school students in Malaysia. The study will be conducted in cooperation with stakeholders (canteen and school convenience shop operators) in secondary schools. The specific objectives are:

- To test the feasibility of providing healthier food options (such as minimising SSBs, more availability of fruits and vegetables) at the school canteen in cooperation with food vendors.
- To test the feasibility of assessing changes in food choices to healthier options while at school among adolescents pre- and post-intervention.
- To test the feasibility of assessing changes in anthropometric measurements of adolescents, pre- and post-intervention.

## Methods

### Overview of Study Methodology

This study is part of the MyHeARTBEaT project (IF017-2017) approved by Research Ethics Committee at the University of Malaya Medical Centre (MREC ID NO: 2018214-6029). The study was registered at the ISRCTN registry: ISRCTN 89649533. The trial design is a three-arm, parallel-group, un-blinded, feasibility cluster randomized controlled study undertaken within six schools in Selangor and Perak states in Malaysia. It will compare two intervention arms (1 and 2) against a usual practice control conducted in six secondary schools, of which four schools will receive the interventions and two will serve as controls. Two interventions are proposed which will focus on providing healthier food options available for sale at the school canteen and convenience shops in the selected schools. Intervention 1 will entail training the canteen and school convenience shop operators to prepare healthier food options in the canteen. The research team will train food operators on the benefits of selling healthy food. Intervention 2 also will create awareness, with the cooperation of food vendor operators among students on consuming healthy food. It will include subsidising the price of vegetables, fruits, and low energy-dense *kuih* (traditional cake). The training of the canteen and school convenience shop operators will follow intervention (1) and focus on training the staff who will sell subsidised fruits, vegetables, and low-energy *kuih*. The control group will continue to sell the usual food.

**Figure 1:**
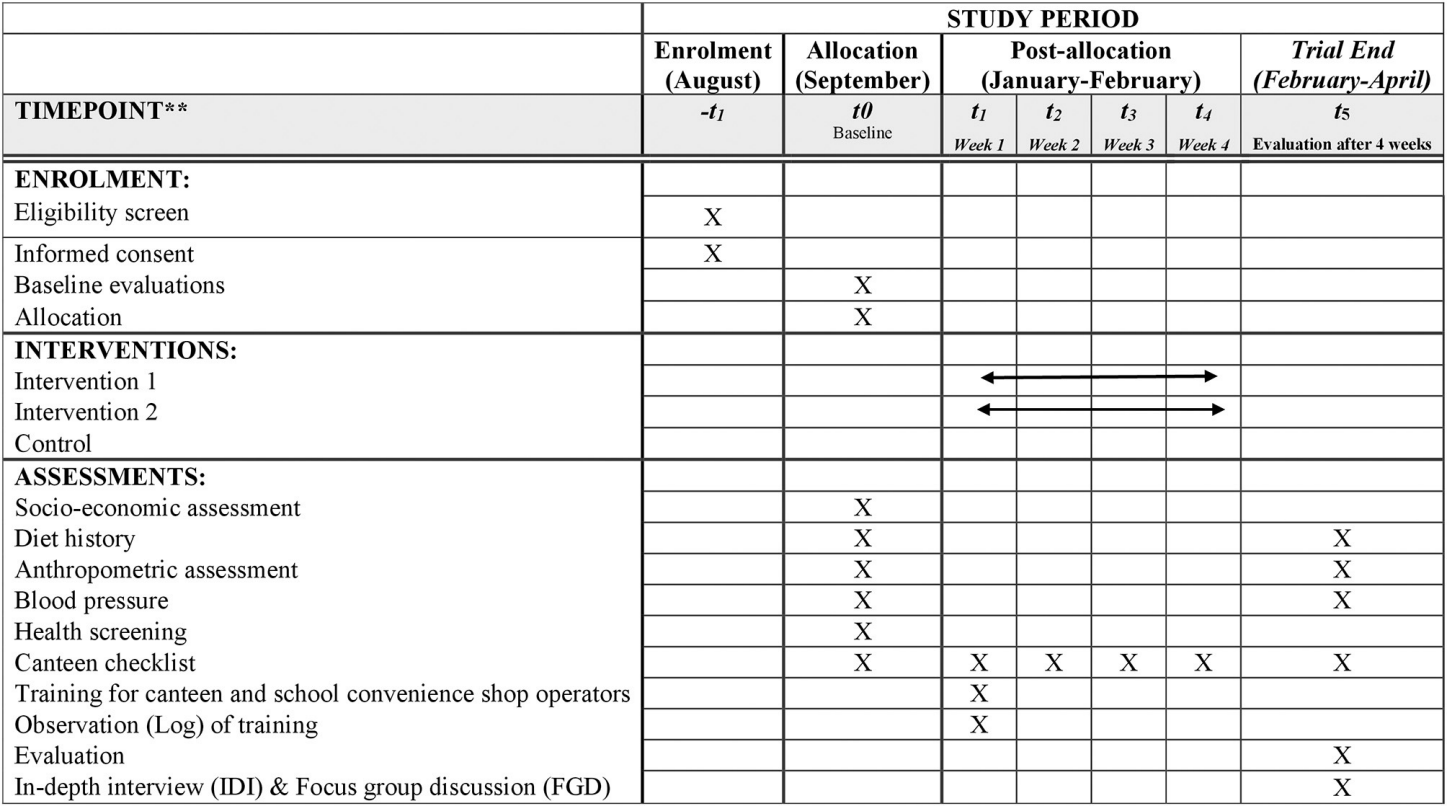
Summary of enrolment, intervention and assessment procedures based on the SPIRIT figure.

**Figure 2:**
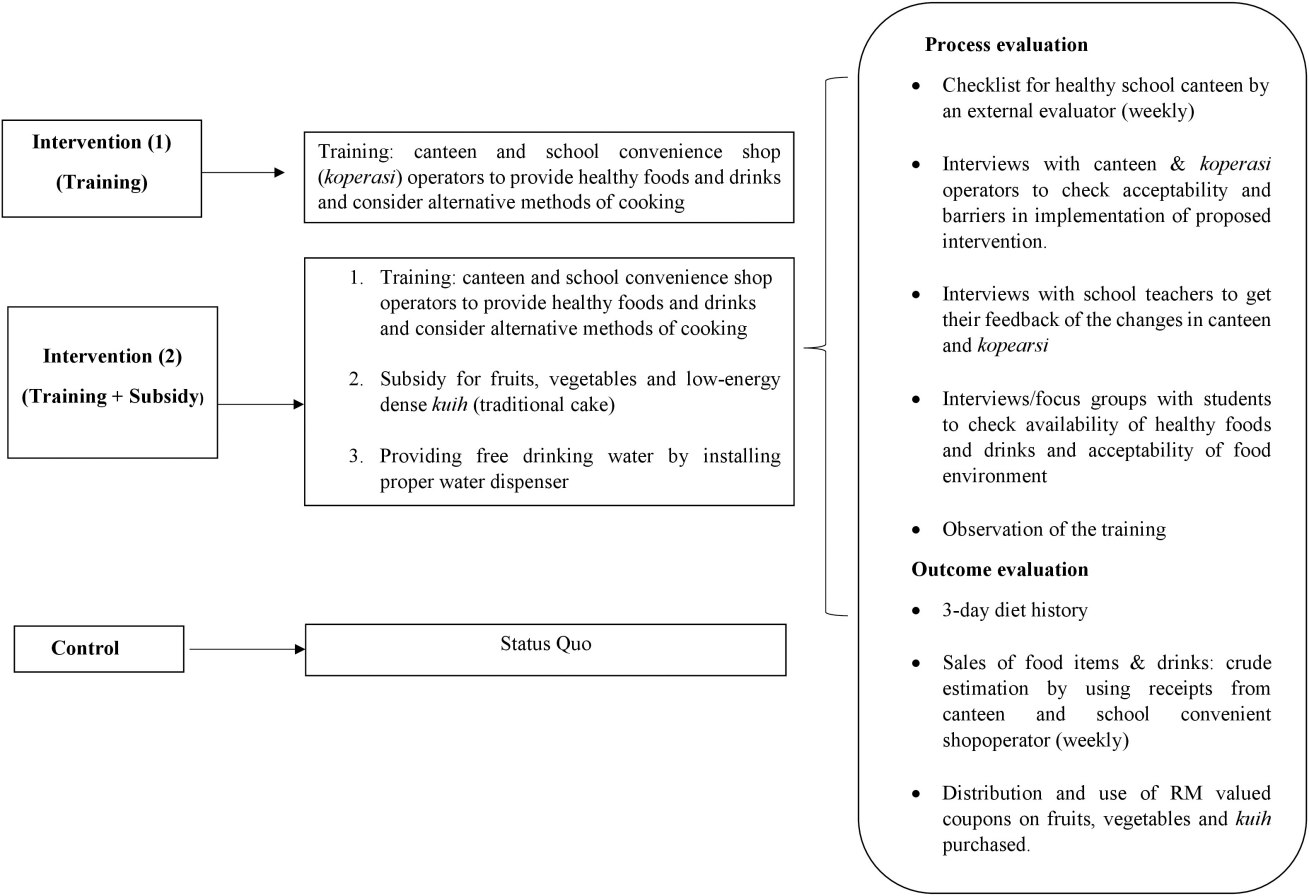
Logic model of intervention.

Trained dietitians will audit the canteen menu and food items sold by the school canteen and convenience shops in all schools (during the 4-week intervention phase, once a week). The menu audit process has been implemented in other studies (45). The study outcome measures will be assessed at the individual level and will consist of a 3-day diet history assessment and anthropometric measurements that will be conducted at baseline and post-intervention (4 weeks after intervention). Focus group discussions with students who will be involved in the intervention and interviews with teachers, school headmasters, and canteen operators will be conducted post-intervention to explore the acceptability and barriers in implementing the proposed intervention (see Supplementary Material 1). Estimation of sales will be done based on weekly receipts from the canteen and school convenience shop operators of healthy food items, such as vegetables, fruits, and low-calorie *kuih*. The data will be collected at baseline (August to September 2018) and the following intervention will be carried out after 3 months the year after (January to April). The summary of intervention assessment and logic model are shown in Figures 1, 2, respectively.

### Sample Size Estimation

The National Institute for Health Research guidelines indicated that no formal power calculation is required for feasibility study (46). As this study will be a feasibility study, a formal power calculation based on identifying evidence for effectiveness will not be performed and no sample size calculation will be undertaken. The sample size of six schools (four intervention and two control arms) will be grouped based on the minimum recommended for a pilot cluster randomized trial (47).

Based on recent experience in local secondary schools, we predict that between 75 and 80 students from each school will reach the age of 15. Therefore, we assume that the sample size will range between 450 and 480 adolescents. This is a pragmatically chosen sample to detect the feasibility evidence, recruitment rates, and any barriers to implement the research methodology. Feasibility study is conducted to determine the necessary sample size needed to evaluate the intervention. It is premature to specify the required sample size for a future trial, however it is useful to get a broad indication of the estimated sample size for the trial. Thus, the sample size for this study will be chosen to provide a sufficient number of schools and students to test each of the three conditions in urban and rural areas and provide an indication of the likely sample size for a full trial.

### Recruitment and Randomisation Procedures

The sample will consist of six schools in Selangor and Perak states in Malaysia as the rural and urban areas, respectively. The sample of six schools will be randomly selected from a list of secondary schools that had previously participated in the MyHeARTs cohort study (48). The previous cohort study followed a stratified sampling design. First, a complete list of the public secondary schools in the selected regions was obtained from the Ministry of Education (MOE) in Malaysia and used as the sampling frame. The schools were classified as urban and rural-based on the criteria provided by the Department of Statistics Malaysia. A total of 15 schools from a cohort that was consisted of eight urban and seven rural schools was chosen as the study sampling frame (48).

The six schools in this study will be first randomly selected from 15 schools, and then the schools will be randomly allocated to the intervention 1, 2, and control arms (two schools per arm). Two schools will be allocated to the intervention 1 group and will receive training only, while two other schools will receive training and subsidies on healthy foods. Another two schools will make up the control group without any involvement in the interventions. However, the control groups will receive manual on healthy canteen at the end of the study. The random selection and allocation of the six schools from the urban and rural locations and division to intervention and control arms will be performed (computer-generated allocation) by an independent statistician from the University of Bristol who will be blind to the school identity. The primary sampling units will be six schools and the secondary sampling will include all 15 years old students of six selected schools who will be invited.

Participants will be recruited from six government-funded secondary schools located in the Selangor (urban) and Perak (rural) areas in Malaysia (three schools from each area). Eligible participants will be Malaysian adolescents (15 years of age) from “secondary three class” (known as “Form three”). The students and their parents or guardians will receive written information about the study as well as consent form 1 week before conducting the intervention and then will be requested to submit the completed consent form next day if possible. The participants will have to speak and write the national language (Malay). Students from religious and vernacular schools will be excluded from the sample since most of them tend to be from a mono-ethnic group.

### Intervention Development

The intervention was developed based on data obtained from systematic reviews (21, 49), reports of the MyHeARTs cohort study (48), and a related qualitative study (50). The findings suggested that a school canteen intervention had merit. The qualitative study was also useful to operationalise the intervention content (50). Focus group discussions with selected adolescents aged 15 years were conducted to understand the available canteen food. The key informants were school headmasters and canteen operators who were interviewed on the dietary habits of students to develop a priority list for intervention (50). All transcripts were analysed and coded. The themes on healthy eating suggestions were availability of healthy options, subsidising healthy foods, and health education and training (50). Stakeholders thought that adolescents' misperceptions, affordability, unhealthy food preferences, and limited availability of healthy options were important barriers preventing healthy eating at school. Furthermore, affordability was a major problem for adolescents in rural schools. Stakeholders perceived that a future school-based intervention might improve the availability and subsidies for healthy foods (50). The intervention was developed and its components guided by the Theoretical Domains Framework for use in behaviour change (51). The research team then mapped potential behaviour change techniques (implementation strategies) to the identified barriers, which will be refined based on considerations of feasibility, potential impacts and context. As a result, two implementation strategies formed the intervention.

### Intervention Overview

This study will explore the feasibility of conducting a dietary intervention in different types of schools (urban or rural) and its potential (evidence of promise) for improving healthy eating practices in adolescents from different socioeconomic backgrounds. It will focus on offering healthy food and beverage options in the school canteen and convenience shop. School canteens will be supposed to support healthy eating based on the healthy canteen guidelines designed by the Ministry of Education (MOE) (52). This will be achieved by increasing the availability and promotion of an extensive range of foods that should make up the majority of a healthy diet (GREEN). AMBER foods can be consumed only sometimes, choosing healthier alternative foods and avoiding large serving sizes that should be eaten in moderation. RED foods are “stop and think foods” that do not meet specified minimum nutrient criteria (RED). The intervention will try to overcome barriers to healthy canteen guidelines implementation and adherence. Specifically, the intervention will assist schools to remove “red-flagged” and “ban” food items from the canteen menu while increasing the availability of “GREEN” menu items. The objective will be to ensure more than 50% of all canteen menu items are in the “GREEN” category. Examples of “GREEN”, “AMBER,” and “RED” food items are indicated in Table 1.

**Table 1:** Food classification description.

	**Everyday foods– “GREEN”**	**Select carefully– “AMBER”**	**Occasional foods– “RED”**
Nutritional quality	• High in nutrients and fibre. • Low in saturated fat and/or added sugar and/or salt.	• Moderate in added fat and/or sugar. • Contribute to excessive kilojoules if consumed in large serving size.	• Very low in nutritional value. • High in saturated fat and/or added sugar and/or added salt.
Foods	• Breads and breakfast cereals (those high in fibre and low in saturated fat and sugars). • Fruits and vegetables. • Reduced-fat dairy products. • Lean meat, fish and poultry. • Eggs, nuts, and legumes. • Commercial vegetable-based soups.	• Full fat dairy products. • Processed meats (e.g., corned beef, chicken loaf). • Commercially produced hot foods (e.g., fried rice, pasta, lasagna, and soup dishes). • Reduced-fat/salt commercially produced pastry based or crumbed hot foods (e.g., meat pies and sausage rolls). • English muffin or pita bread-based pizza. • Margarines, oils, spreads sauces, gravies, dressing and mayonnaise. • Snack food bars. • Un-iced and/or reduced-fat and/or fruit-based cakes, muffins, sweet biscuits, slices. • Savoury snacks foods and biscuits. • Low/reduced-fat ice cream and dairy desserts. • Ice blocks/slushies made with 100% juice. • Breakfast cereals (those refined with added sugar). • Savoury commercial products, snack food bars and biscuits.	• Pastry based or crumbed hot foods (e.g., meat pies, sausage rolls). • Processed meat such as salami (unless reduced-fat/low salt). • Fried foods. • Commercial pizza products. • Savoury snack foods–crisps, chips, biscuits or similar products. • Ice-creams and dairy desserts. • Iced cakes, muffins, sweet biscuits, slices. • Cream, butter and chocolate-based spreads.
Drinks	Water. 99% fruit juice in 200 mL serves or less.	99% fruit juice in serves >200 mL but <300 mL.Artificially sweetened drinks.	All sugar-sweetened drinks with >300 kJ per serving and/or >100 mg of sodium per serve are banned from sale in school canteens.

### Intervention Components and Implementation

#### Intervention (1) (Training Only)

Training: canteen and school convenience shop operators will be requested to provide healthy foods and drinks and consider alternative methods of cooking by using a training manual (canteen and school convenience shop operators), which will be developed based on the Malaysian Healthy Canteen Guidelines (52). Canteen managers and staff will be invited to attend a 1-h training workshop to educate them on healthy eating policy, nutrition, canteen stock, and change management. They will also be provided with a “Healthy Canteen Booklet” and will be trained in choosing healthy cooking methods, such as steaming and grilling.

#### Intervention (2) (Training + Subsidy)

The intervention will intend to increase the (i) intake of fruits and vegetables and (ii) consumption of healthy and lower energy *kuihs* (e.g., *keladi rebus (steamed yam), apam (pancake)*, and non-sweetened beverages (e.g., water), rather than high energy-dense snacks and drinks (measured by their sales receipts, dietary intake, coupons).

➢ Training: Canteen and school convenience shop operators will have to provide healthy food options and consider alternative methods of cooking by using the Training Manual developed based on the Malaysian Healthy Canteen Guideline (52). A trained dietitian who is the main investigator of this study (HM) will deliver the training in these two schools, and the canteens will be assessed each week on the foods they sell.
➢ Fruits, vegetables, and low energy-dense (low ED) *kuih* (traditional cakes) will be subsidised. The canteen operators in this group of interventions will receive a weekly allowance (the amount will be modified when necessary) to sell fruit, vegetables, and low ED *kuih* (e.g., *Ketayap, Cakoi, Cucur cempedak, Apam*) at subsidised prices.
➢ All students (Form three students) will receive (Ringgit Malaysia RM 2) coupons that subsidise the price of fruits (will provide 2 days weekly over 4 weeks) and low ED *kuih* (given 2 days weekly over 4 weeks). Therefore, the total value of coupons per week for one student will be RM 4. This coupon will be given to the students weekly. When they buy fruit/*kuih*, the coupon will be placed in a specific box, and the teacher in charge will collect them daily and keep a record of how many coupons have been used as the subsidy.
➢ In the subsidised group, schools will be provided with funds to prepare healthy food. This will give them additional money for their weekly purchases to prepare extra vegetables for the study participants. For example, if they purchase vegetables worth 200 Ringgit (RM) per week, they will get 100 Ringgit (RM) subsidy so that they can prepare an extra tray of vegetables per week. The dietitian will visit the school on a weekly basis for evaluation. The receipts of the vegetables will be given to the dietitian, who will monitor them by visiting the school every week to examine the report by the teacher in charge.
➢ Providing free drinking water by installing a proper water container/tank to dispense drinking water. The dietitian will check every week whether the water will be filled up during the school period and whether it will be utilised by the students. This will be recorded by the teacher in charge and cross-checked during the weekly visit of the dietitian.

#### Control

The control group will consume the food are typically available at the school canteen and convenience shop. No intervention will be made in terms of their food choices as well as no advice nor resources will be provided during this intervention period. The canteen and convenience shop operators will have their usual service without changes at two schools, which will be considered as the controls. The control school will receive usual healthy canteen guidelines as given by the Ministry of Health.

### Data Collection

Data will be collected at baseline and post-intervention. All six eligible governmental schools with an operational canteen will be required to complete baseline data collection. The schools' characteristics, including school size, location, and the number of days the canteen operates, will be captured before randomisation. Furthermore, canteen vendors will be asked to show the serving menu that captures all foods (even snacks and beverages) available for sale at school canteens to the research team before the canteen checklist assessment.

### Dietary Assessment

A questionnaire will be used to obtain information regarding adolescents' dietary intake. This study will use a validated 3-day diet history to ensure greater accuracy, namely to estimate the respondents' energy intake (16). The tool was pre-tested on 40 participants from two different schools (one school each from rural and urban areas, respectively) (16). Qualified and trained dietitians will conduct an open-ended interview with adolescents to collect information on their food habits through the day (breakfast, mid-morning snacks, lunch, afternoon tea, dinner, and supper). A flip chart will be used as an additional tool to assess the subjects' diet and help estimate their portion size. Nutritionist Pro™ Diet Analysis software will be used to assess the nutritional content of the food (16).

Any misreporting may be heightened in dietary intervention studies. A reliable and valid short form of the Marlowe-Crowne social desirability scale will be administered to identify and control participants prone to these traits (53).

### Anthropometric Measurements

Height will be measured using a vertical stadiometer (Seca Portable 217, Seca, UK) and will be recorded to the nearest millimetre or one-tenth of a centimetre (0.1 cm). Body weight will be assessed using a digital, electronic weighing scale (Seca 813, Seca, UK). It will be based on the nearest one-tenth of a kilogram (0.1 kg). Body fat composition will be evaluated using a portable body composition analyser (Tanita SC-240 MA, the Netherlands). Waist circumference (WC) and hip circumference (HC) will be measured using a non-elastic Seca measuring tape (Seca 201, Seca, UK), to the nearest millimetre.

### Blood Pressure

The help of two qualified medical officers will assess the blood pressure and pulse rate of the participants. The participants will seat in the upright position with their right upper arm position at the level of the heart with both feet flat on the floor. Systolic and diastolic arterial blood pressure will be obtained using a stethoscope and a mercury sphygmomanometer.

### Outcomes

#### Potential Trial Outcomes

As this is a feasibility study, the focus of the current study will be whether the intervention can be delivered as intended and desired outcomes can be attributed. It is envisaged that if we have to move to a larger trial that the primary outcome will be the participants' consumption of healthy canteen items. The participants will be deemed as consuming healthy canteen items or green foods based on their dietary intake of more than 50% of listed menu items. The canteen operators will be tasked with auditing the green items available for sale at the school canteen. During follow-up, two dietitians will audit the results based on the canteen audit and discussion with canteen staff. Food classification tools and guidelines published by the Ministry of Education in Malaysia will be used to categorise the food (see Table 1).

#### Secondary Trial Outcomes

The secondary outcome of the trial will be changes in the dietary intakes and nutritional status of adolescents at school.

### Process Evaluation of the Intervention

Two methods will be used for process evaluation of the intervention, namely external and internal audit using a checklist and qualitative assessment.

#### External and Internal Audit

The process of evaluation will include a checklist for Healthy School Canteens by dietitians to check the availability of healthy food and drink options, cooking with less oil or less salt, preparing more low energy-dense *kuih*, putting extra vegetables in some dishes and ensuring they provide fewer foods from the amber group and more foods from the green group. Furthermore, there will be an observation (log) of the training for the canteen and school convenience shop operators. The Healthy School Canteen Checklist will comprise five sections; two sections on the vendor and school information (section A and B), three evaluation sections where the basic criteria will be evaluated for healthy dishes (section C), healthy food serving method (section D), and traffic light method (section D). Each of the three evaluation sections will carry a percentage for a total score of 100%. Section C will account for 45%, while sections D and E will account for 20 and 35% of the assessment scores (see Supplementary Material 2). The assessment scores must be ≥80% to be recognised as a healthy cafeteria based on MOE Healthy Canteen Guidelines.

The dietitians will have access to the canteen, observe, and check, and they will ask questions about cooking methods. Before the evaluation period using the Healthy School Canteen Checklist, two external dietitians will be trained to ensure consistency in data evaluation. Each external dietitian will be independently assigned to evaluate the same intervention schools (canteen and school convenience shop vendors) over the 4-week period. Any discrepancies arising between two qualified and trained dietitians will be resolved via discussion between the two dietitians, when an agreement could not be reached, a discussion will be held with the principal investigator.

#### Qualitative Assessment

The use of qualitative research methods is essential to gain insights into the implementation process and context and participants' perspectives on the intervention. Qualitative methods will be used to assess the acceptability and feasibility of the interventions, as well as the study procedures. Interviews with canteens and school convenience shop operators will be conducted to explore the acceptability and barriers in the implementation of the proposed intervention, and their understanding of healthy and unhealthy foods based on the traffic light concept. A total of 12 participants, one canteen operator and one school convenience shop operator per intervention school will be asked about any changes in cooking methods after the training sessions and any changes in their knowledge and attitudes toward preparing healthy foods, which should be reflected in their cooking practices. In addition, focus groups will be conducted with adolescents to explore their opinions on the availability of healthy food and acceptability of the food environment. A total of 80 students will participate in focus groups, 20 per intervention school (10 boys and 10 girls), and they represent the major ethnic groups in Malaysia (Malay, Chinese, Indian). Interviews with school teachers (*n* = 12) will also be conducted to obtain their feedback for the changes at canteens and school convenience shops. The interview guide is as attached in the Supplementary Materials.

### Planned Analysis

Feasibility studies are not designed to evaluate the effectiveness of interventions (54). As this is a feasibility study, no formal comparison between the study arms will be undertaken and no significance testing will be conducted. Descriptive statistics such as the mean (standard deviation) for continuous variables and frequency and proportions (*n*, %) for categorical variables will be used to evaluate the research hypotheses. In light of the feasibility study and the associated lack of statistical power, all effects will be evaluated based on effect size and *p*-values will not be reported. The analysis will be reported using the relevant CONSORT checklists (55). For quantitative data analysis, the SPSS software (Version 25.0, Chicago, IL, US) will be used. All focus group discussions and interviews will be audio-recorded, transcribed verbatim and entered into NVivo software to analyse quantitative data and facilitate data coding. Field notes and memos will be also analysed and the framework approach for qualitative data analysis will be used (56).

### Participant Confidentiality

All study personnel will be trained in the requirements to ensure the participants' confidentiality. The principal investigator will protect the confidentiality of participants' information. Participant identification code will be assigned upon study enrolment. The data will be kept centrally by the principal investigator and will be anonymised and identified by a unique code. Moreover, access to personally identifiable information will be limited to members of the research team. Documents that will not be anonymised, such as signed informed consent forms will be held by the principal investigator in a locked cabinet in the department at the university.

## Discussion and Conclusion

The MyHeARTBEaT study proposes the design and rationale for a school-based intervention to facilitate the adoption of a healthy canteen policy in Malaysian schools. The study will try to determine if the audit and subsidisation for healthy foods can improve/increase the availability of the healthy food and drinks option at the school canteen and convenience shop, improve the number of school canteens, which offer healthy food options, increase the number of students who consume greater amounts of fruits and vegetables, and decrease the intake of SSBs by students. This strategy may have the potential to offer cost-effective and sustainable support to improve the school canteen food environment. The insights into the effects of price-related approaches to improve food consumption and purchasing behaviours may provide evidence to build appropriate policy and programme responses to the epidemic of obesity and poor diet quality currently facing Malaysian adolescents (49).

Limited healthy lifestyle school-based interventions were conducted in Malaysia. As such, this study addresses a significant research gap and a potentially valuable missed opportunity in efforts to promote healthy eating behaviours among Malaysian adolescents. The research may contribute to the literature on the adverse effects of poor nutrition and associated adverse health outcomes, including obesity, in the Malaysian population and worldwide. While the impacts of school-based canteen interventions have been examined in other studies in Malaysia, the upcoming trial will be the first to analyse the impact of healthy food subsidisation in Malaysian school canteens and targeting both school convenience shops and canteens for intervention. The intervention will be considered to warrant evaluation via a full trial if the study achieves more than 50% of all healthy canteen menu items in the “GREEN” category and recruits 80% of eligible participants. Based on this feasibility, we shall plan either to use interventions such as training of canteen and school convenience shop operators only (intervention 1) or training with some financial subsidy (intervention 2) for the full trial.

This school-based study is intended to provide new insights regarding the implementation of healthy foods and drinks environment in Malaysian schools, including distribution of subsidised fruits and vegetables and low ED *kuih* that may improve healthy food intake among Malaysian adolescents. The possible implication of this study includes minimizing the availability of SSBs sold in school canteen in the future. We also want to see the availability of fruits and vegetables at the school canteen and the acceptance of healthier food options among the school adolescents. By offering more of healthier option of foods and beverages in school, it will be good to evaluate if changes toward environment may enable the students for better healthy options. From this healthy canteen and convenience shop interventions, we would like to see any changes on anthropometric measurements within a month of intervention. This will enable us to plan for larger scale study in the future. With all the intervention planned, it is anticipate it should be acceptable as we do take into account the diversity of Malaysian ethnic background.

This study is anticipated to be useful in intervention development process and identifying ways to improve the availability of healthy foods within the school environment and modifying healthy canteen guidelines and policy development. It is expected that the findings from this study can provide a framework for the conception and implementation of nutritional interventions at the school canteens in Malaysia that will be tested for efficacy in a future definitive trial.

## Ethics Statement

Ethical approval was given by the Medical Ethics Committee at University of Malaya Medical Centre, reference number 2018214-6029. Written informed consent to participate in this study was provided by the participants' legal guardian/next of kin.

## Author Contributions

HM, SM, and TS conceptualised the study and drafted the initial study protocol. SM, HM, TS, AP, ZT, MD, MJ, MA, and RJ participated in the design of the protocol. All authors critically reviewed the draft of the manuscript and approved the final version.

## Conflict of Interest

The authors declare that the research was conducted in the absence of any commercial or financial relationships that could be construed as a potential conflict of interest.
