# Supplementary material for: School-Based Intervention to Improve Healthy Eating Practices Among Malaysian Adolescents: A Feasibility Study Protocol
Source: Front Public Health. 2020 Sep 22;8:549637. doi: 10.3389/fpubh.2020.549637 (PMC7536333; doi:10.3389/fpubh.2020.549637)
Supplement: Supplementary file 1 [file Data_Sheet_1.PDF]

# *Supplementary Material 2*

## Healthy Canteen Checklist (Modified version of Malaysian MoH checklist)

**Section A: Employer information**

1. **Name** : ……………………….
2. **No. Identification card** : ……………………….
3. **No. Tel** : ……………………….
4. **E-mail** : ……………………….

**Section B: Place Information**

1. **Name and address of School** : ………………………..
2. **Name of company** :……………………..
3. **Operating Time:** **Time:** ……….. **Day:** ………..

Criteria for Healthy Dishes at the Canteen (adapted from Ministry of Health, Malaysia)

| **Item** | **Criteria** | **Type of Dishes** | **Notes** |
|---|---|---|---|
| **Cereals, Grains and Tubers** | 1) At least 2 choices of grains<br>2) At least one dish is a source of fibre<br>3) If does not offer whole grains for at least one dish, 1 legume dish should be made available. | 1) Bread /Chapati/ Naan<br>2) Cereals/ Oats<br>3) Pasta/ Noodles<br>4) Rice/ Brown Rice<br><br>**High fibre options:**<br>1. Wholemeal bread<br>2. Chapatti<br>3. Brown rice<br>4. Oats | |
| **Fruits & Vegetables** | 1) Offers at least 3 types of vegetables and 3 types of fruits (not inclusive of garnishes, deep-fried vegetables and creamy soups with vegetables such as mushroom soup or salads mixed paste, mayonnaise or Thousand Islands dressing<br><br>2) Only one vegetable dish cooked with coconut milk is allowed | 1. Vegetable soup, stir-fried or boiled vegetables.<br>2. Fruits<br>3. Fruit salad<br>4. 100% fruit or vegetable juice<br>5. Tomato-base pasta sauce<br>6. Vegetable toppings for pizzas<br>7. Vegetable salad or raw vegetable servings | Dressings for salads and fruit 'rojak' should be served separately. |
| **Fish/ Chicken/ Eggs/ meat or Meat Alternative. Tempeh soya product) ( Method of cooking)** | At least half of the dishes are cooked in a healthy way or using a healthy recipe | Examples:<br>1. Ikan Asam Rebus (Fish stewed with tamarind)<br>2. Roasted chicken<br>3. Steamed fish<br>4. Ikan pindang (fish cooked using boiled method)<br>5. Baked fish<br>6. Fish/ Chicken/ meat curry without coconut milk<br>7. Tofu soup<br>8. Roasted tofu<br>9. Steamed tofu<br>10. Boiled eggs | |
| **Milk and dairy products** | 1) Low-fat milk or skimmed milk is provided<br>2) Evaporated milk/creamer is provided upon request only<br>3) Non-dairy creamer is not allowed | 1. Low-fat milk<br>2. Skimmed milk<br>3. Low fat yoghurt | |
| **Desserts** | At least half of the dishes use a healthy method of cooking or uses a healthy recipe. | 1. Popiah basah (non-fried spring rolls)<br>2. Apam kukus (steamed Apam)<br>3. Steamed cakes<br>4. Stuffed tofu<br>5. 'Kuih kukus' ( steamed cakes) | |

**Section C: Basic criteria of the cafeteria (45%). Refer to the above description.**

<table>
<tr><th rowspan="2">No.</th><th rowspan="2" colspan="2">Title</th><th colspan="3">Mark √ in space most appropriate</th><th>Notes</th></tr>
<tr><th>Yes</th><th>No</th><th>N/A *</th><th></th></tr>
<tr><td rowspan="4">C1<br>Rice, Noodles, Bread, Cereals, Grain & Tubers</td><td>i.</td><td>Has at least 2 food choices of grains</td><td></td><td></td><td></td><td></td></tr>
<tr><td>ii</td><td>The fried food is not greasy</td><td></td><td></td><td></td><td></td></tr>
<tr><td>iii</td><td>At least one dish is a source of fibre</td><td></td><td></td><td></td><td></td></tr>
<tr><td>iv</td><td>If whole grain is unavailable, there should be at least one dish with legumes</td><td></td><td></td><td></td><td></td></tr>
<tr><td rowspan="5">C2<br>Vegetables</td><td>i</td><td>Serving at least 3 types of vegetables, not inclusive of garnishes, deep-fried vegetables and creamy soup (like creamy mushroom soup) and salad mixed paste, mayonnaise or thousand islands</td><td></td><td></td><td></td><td></td></tr>
<tr><td>ii</td><td>Only one vegetable dish cooked with coconut milk is allowed</td><td></td><td></td><td></td><td></td></tr>
<tr><td>iii</td><td>Vegetables should not be cooked too long</td><td></td><td></td><td></td><td></td></tr>
<tr><td>iv</td><td>The fried vegetables are not greasy</td><td></td><td></td><td></td><td></td></tr>
<tr><td>v</td><td>Vegetables are not cut out very small</td><td></td><td></td><td></td><td></td></tr>
<tr><td rowspan="2">C3<br>Fruits</td><td>i</td><td>Offer at least 3 types of fruits</td><td></td><td></td><td></td><td></td></tr>
<tr><td>ii</td><td>Separate salt, sour powder, ‘petis’ and soy sauce</td><td></td><td></td><td></td><td></td></tr>
<tr><td rowspan="3">C4<br>Fish/Chicken/Eggs/ Meat or Meat alternative</td><td>i</td><td>At least half of the dishes provided are cooked in healthy way….boiled /steamed/roast / baked methods.</td><td></td><td></td><td></td><td></td></tr>
<tr><td>ii</td><td>Provide non-greasy fried dishes</td><td></td><td></td><td></td><td></td></tr>
<tr><td>iii</td><td>Add vegetables in cooking</td><td></td><td></td><td></td><td></td></tr>
<tr><td>C5<br>Legumes</td><td colspan="2">Serving at least 1 type of legume-based dishes. (e.g. pine nuts, peanuts, tempeh, tofu, pastry, vadai)</td><td></td><td></td><td></td><td></td></tr>
<tr><td>C6<br>Milk & dairy products</td><td colspan="2">• Provide a low-fat or skimmed milk drink or sell low-fat/skim milk<br>• Evaporated milk/creamer is provided only upon request<br>• Non-dairy creamer is not used</td><td></td><td></td><td></td><td></td></tr>
<tr><td>C7<br>Desserts</td><td colspan="2">At least half are healthy dishes</td><td></td><td></td><td></td><td></td></tr>
<tr><td colspan="3">Total √</td><td></td><td></td><td></td><td></td></tr>
<tr><td colspan="3">Percentage value:<br>percentage points = $\frac{\text{Total } \surd \text{ yes}}{\text{Total} \surd \text{ [ yes + No]}} \times 45\%$</td><td colspan="4">Percentage points = $\frac{\square}{\square} \times 45\% = \square$</td></tr>
</table>

***N/A: Not applicable**

# Traffic light summary table

| BANNED DRINKS ✗ All drinks previously categorised as RED can no longer be sold in school canteens and vending machines as of Term 1, 2007. | AMBER DRINKS 'Select Carefully' | GREEN DRINKS 'Fill the Menu' |
|---|---|---|
| Drinks with more than 300kJ per serve or more than 100mg sodium per serve. Check:<br>• soft drinks<br>• energy drinks<br>• fruit drinks<br>• flavoured mineral waters<br>• sports drinks<br>• cordials<br>• iced teas<br>• sweetened waters<br>• sports waters<br>• flavoured crushed ice drinks | • diet soft drinks<br>• full fat plain milk<br>• full fat flavoured milk<br>• full fat soy drinks<br>• 99% fruit juices – 300ml serve size or less<br>• sugar sweetened drinks with less than 300kJ per serve and less than 100mg of sodium per serve<br>• sugar sweetened frozen crushed ice drinks with less than 300kJ per serve and less than 100mg of sodium per serve | • water<br>• reduced fat plain milk<br>• reduced fat flavoured milk<br>• reduced fat soy drinks<br>• 99% fruit juices – 200ml serve size or less<br>• high fibre fruit juices – 250ml serve size or less<br>• 99% fruit juice frozen crushed ice drinks – 200ml serve size or less |

Note: "Serve size" means the size of a food or drink as sold in the school canteen

| | |
|---|---|
| GREEN<br>Always on the canteen menu | Foods and drinks categorised as GREEN are the best choices for the school canteen menu as they contain a wide range of nutrients and are generally low in saturated fat and/or sugar and/or sodium (salt)<br>These foods and drinks should be:<br>• Available every day<br>• Included as the main choices on the canteen menu<br>• Presented in attractive and interesting ways<br>• Promoted as tasty, good value choices |
| AMBER<br>Select Carefully | Foods and drinks categorised as AMBER contain some valuable nutrients as well, but may also contain higher levels of saturated fat and/or sugar and/or sodium (salt). If eaten in large amounts these foods may contribute to excess energy (kilojoules) being consumed.<br>These foods and drinks should be:<br>• Sold in smaller serve sizes<br>• Less prominent on the canteen menu<br>• Moved towards the 'GREEN' end of the spectrum at every opportunity |
| RED<br>Not recommended on the canteen menu | Foods and drinks categorised as RED are low in nutritional value and may contain excess energy (kilojoules) and/or saturated fat and/or sodium (salt) and/ or sugar<br>These foods and drinks should:<br>• Not be provided in the healthy school canteen |

**Section D:** Healthy Food Serving Method (20%)

| No. | Things | Mark √ in space most appropriate | | | Notes |
|---|---|---|---|---|---|
| | | Yes | No | N/A* | |
| **D1** | More vegetables are added in | | | | |
| **D2** | Do not use MSG/stock instant | | | | |
| **D3** | Extra sauce / soy sauce / salt / parsley is not prepared at the dining table | | | | |
| **D4** | Food/drink is not too sweet | | | | |
| **D5** | Food is not too salty | | | | |
| **D6** | Providing less sugar beverages at the request of the customer | | | | |
| **D7** | Also sell / provide drinking water | | | | |
| Total √ | | | | | |
| Percentage value: percentage points = $\frac{\text{Total √ yes}}{\text{Total√ [ yes + No]}} \times 20\%$ | | Percentage points = $\frac{\square}{\square} \times 20\%$ $\square$ | | | |

***N/A: Not applicable**

**Section E:** Traffic light Method (35%)

| No. | Things | Mark √ in space most appropriate | | | Notes |
|---|---|---|---|---|---|
| | | Yes | No | N/A* | |
| **E1** | Are green drinks the main drinks on offer in the canteen | | | | |
| **E2** | Are green foods the main foods on offer in the canteen | | | | |
| **E3** | Does the canteen limit the number of 'amber' drinks in the menu? | | | | |
| **E4** | Does the canteen limit the number of 'amber' foods in the menu? | | | | |
| **E5** | Any foods from RED category? | | | | |
| **E6** | Any drinks from RED category? | | | | |
| Total √ | | | | | |
| Percentage value: percentage points = $\frac{\text{Total √ yes}}{\text{Total√ [ yes + No]}} \times 35\%$ | | Percentage points = $\frac{\square}{\square} \times 35\%$ $\square$ | | | |

***N/A: Not applicable**

**Section F:** Overall (%)

Overall (Section C+D+E)

Healthy cafeteria: Cafeteria recognition: Assessment scores must be ≥ 80% to get a healthy cafeteria's recognition
