# Supplementary material for: School-Based Intervention to Improve Healthy Eating Practices Among Malaysian Adolescents: A Feasibility Study Protocol
Source: Front Public Health. 2020 Sep 22;8:549637. doi: 10.3389/fpubh.2020.549637 (PMC7536333; doi:10.3389/fpubh.2020.549637)
Supplement: Supplementary file 2 [file Data_Sheet_2.PDF]

Supplementary Material 1

Interview Topic Guide (Canteen Operators)

1. What has been your experience of implementing the Healthy School Canteens (HSC) Guideline?
2. Is there anything in the HSC Guideline that you don't understand or doesn't make sense to you?
3. Is the use of the HSC Guideline generally supported by the school community?
4. Does your school have a healthy food policy? If so, how does it fit with the HSC Guideline?
5. Have you called upon any other help or resources to interpret the HSC criteria? Who or what resources have you used? Were they helpful?
6. What other help or resources would be useful and how would you use them?
7. Are there more items categorised as GREEN according to the HSC criteria on the menu now, than before implementation? If yes, please give examples.
8. What steps have you taken to 'GREEN' the items categorised as AMBER on the menu? Have you run any healthy food promotions? If so, please describe.
9. Have you noticed any obvious changes to school canteen sales since the implementation of the HSC Guideline? If so, what are they? Do you think these changes are related to the implementation of the HSC Guideline?
10. Has the implementation of the HSC Guideline led to any unexpected changes (positive or negative) in the school canteen? (e.g. less litter in the school area, students bringing more foods and drinks categorised as RED from home)
11. What has helped in the implementation process?
12. What has hindered the implementation process?
13. Have there been any changes to your canteen or school in the last month that may have had an impact on how the implementation went? (For example, change from paid to volunteer manager or vice versa; major refurbishment; change in governance/organizational arrangements; purchased or received a donation of major equipment).

Anything else you would like to say?

Focus Group Discussion Topic Guide (students)

1. What has been your experience from the implementation of the Healthy School Canteens?

2. Are there more items categorized as healthy food items on the menu now, than prior to implementation? If yes, please give examples.

3. Are you interested of buying healthy options?

4. What foods & beverages do you eat just about every day that you think are healthy? (i.e. –orange juice, it has Vitamin C). From the answer question number 3 would you like to buy the foods and drinks on a daily basis?

5. What foods & beverages do you eat just about every day that you think are not healthy? (i.e.- potato chips & soda). What is the unhealthy food that you consumed daily?

6. What are the challenges that you faced from buying healthy foods at school? After school? (prompt: price…etc)

7. What suggestions would you make to your school to promote healthy eating? What would you suggest to make the school canteen a better place to buy healthy food? Do you like this program?
